# Effects of the *in ovo* injection of an *Escherichia coli* vaccine on the hatchability and subsequent early post hatch characteristics of commercial layer chicks

**DOI:** 10.1016/j.psj.2024.104562

**Published:** 2024-11-22

**Authors:** K.E.C. Elliott, L.L. Lindsey, J.D. Evans, S.A. Leigh, K.J. Robinson, S.A. Fatemi, A. Mousstaaid, P.D. Gerard, J. L Purswell, E.D. Peebles

**Affiliations:** a Department of Poultry Science, Mississippi State University, 810 Highway 12 East, MS 39762, USA; b USDA-ARS, Poultry Research Unit, MS 39762, USA; c School of Mathematical and Statistical Sciences, Clemson University, Clemson, SC 29634, USA

**Keywords:** Body weight, *E. coli*, Growout, *In ovo*, Layers

## Abstract

In the commercial table egg industry, avian pathogenic *E. coli* (APEC) can lead to significant economic loss and bird mortality. The Poulvac *E. coli* vaccine (PECV) (Zoetis, Parsippany, NJ) may be administered to poultry post hatch via coarse spray or in drinking water to protect against APEC infections. The purpose of this experiment was to *in ovo* vaccinate commercial layers with various doses of the PECV to evaluate hatchability and post hatch development of the chicks through 21 d of age. Eggs were either non-injected or vaccinated at 18 d of incubation with a diluent-injected control or one of the PECV treatments which included a full dose (2.65 × 10^7^*E. coli* CFU/egg) or dilutions of the full dose to produce 2.65 × 10^5^, 2.65 × 10^3^, or 2.65 × 10^1^ CFU/egg. Mean hatch of injected eggs was significantly (P < 0.0001) affected by treatment, with all the PECV treatments significantly decreasing hatch. Mean chick BW was determined on d of hatch and at 1, 2, and 3 wk post hatch, and mean chick length was determined at 3 wk post hatch. Chick BW was significantly (all P < 0.0001) different between treatments at 1, 2, and 3 wk of age, with the chicks that had received diluent alone having the highest BW, and the chicks that had received the full dose having the lowest BW. Chick length was significantly (P = 0.045) different between treatments, with the chicks in the full dose treatment having a shorter body length than the chicks in the 2 control groups. Cumulative mortality from 0 to 21 d of growout was 5.41% in the full dose treatment, whereas no mortalities were observed in the other treatment groups. While layer chicken embryos were able to survive to 3 wk post hatch after having received the PECV by *in ovo* injection, the full dose of the vaccine increased cumulative chick mortality and decreased chick BW through 3 wk post hatch.

## Introduction

Avian pathogenic *E. coli* (**APEC**) is a pathogenic strain of *E. coli* that can cause significant economic losses within the table egg industry (Barnes et al., 2008). An infection of APEC can manifest itself in multiple ways, including yolk sac infection, cellulitis, swollen-head syndrome, salpingitis, acute fatal septicemia, peritonitis, airsacculitis, and orchitis (Barnes et al., 2008). *Escherichia coli* is a gram-negative bacterium, and it contains lipopolysaccharide (**LPS**) as a component of its outer membrane (Barnes et al., 2008). It has been found that LPS can cause sickness in laying hens, leading to adverse behavioral manifestations such as lethargy, ruffled feathers, immobility, and low responsiveness (Lieboldt et al., 2016; Liu et al., 2019). Affected birds will normally show clinical signs, such as the inability to eat and drink, as well as problems with their balance and gait (Barnes et al., 2008). Infection in a layer facility can be spread by the inhalation of dust particles (Dho-Moulin and Fairbrother, 1999). It can also be spread through contact with feces (Barnes et al., 2008). Early APEC infections can arise in hatcheries and infections can lead to amplified first week mortality (Dho-Moulin and Fairbrother, 1999; Swelum et al., 2021).

Due to the possible detrimental effects of an APEC infection, vaccination against APEC is currently practiced at the commercial level. The Poulvac *E. coli* vaccine (**PECV**; Zoetis, Parsippany, NJ) can be administered to commercial layer pullets at one d of age and again at 12 to 14 wk of age (Zoetis, 2021a). The current method of PECV application is either via drinking water or coarse spray. Both methods offer protection for broilers as well as layers (Zoetis, 2021a, 2021b, 2021c).

One alternative vaccination method for early immunity is through *in ovo* vaccination. In previous research employing the PECV *in ovo*, containing between 6.5 × 10^2^ and 6.5 × 10^4^ CFU of *E. coli*, Lindsey et al. (2022) observed that in comparison to its injection in the air cell, its injection into the amnion (**AM**) in layer hatching eggs at 18 d of incubation (**DOI**) resulted in a decrease in hatchling BW. In a later study by Fatemi et al. (2023), in which the PECV was injected only into the AM at 18 DOI, it was shown that an increase in *E. coli* dose between 4.4 × 10^2^ and 4.4 × 10^8^ CFU decreased hatchability and hatchling yolk-free BW and body length at 22 d. However, effects of the *in ovo* administration of the PECV on the post hatch characteristics of layer pullets during rearing have not been previously investigated. Both chick BW and body length can be indicators of chick quality (Molenaar et al., 2008), and chick BW can be greatly affected by bacterial infection (Elliott et al., 2018). In a study in which 4 different doses of the Poulvac® Myco F vaccine were administered *in ovo* at 18 DOI, it was found that an increase in dose led to a significantly lowered BW in layer pullets that were reared to 6 wk of age (Elliott et al., 2018). Similar results were found in a study by Montgomery et al. (1999), who, at 12 DOI, injected Ec^NAL^, an adapted form of *E. coli*, into the chorioallantoic sac of broiler embryos. When birds were reared until 21 d of age, it was reported that the mean BW of the chicks that had been injected with Ec^NAL^ were significantly lower than the mean BW of the control groups (Montgomery et al., 1999).

Therefore, the objectives in this study were to not only re-examine the effects of an 18 DOI *in ovo* injection of the PECV, including a lower dose of *E. coli*, on the hatchability of Hy-Line W-36 layer hatching eggs, but to also determine its effects on the subsequent mortality and characteristics of the hatchlings through 3 wk of post hatch age.

## Materials and methods

### Egg incubation and treatment assignments

Eggs were obtained from a 50-wk-old parent flock that had been vaccinated for *E. coli* 4 times between the ages of 6 and 16 weeks. The eggs were set in NatureForm NMC 1080 incubators (NatureForm Hatchery Systems, Jacksonville, FL) which served as both setter and hatcher units for the study. The eggs were randomly divided into 6 treatment groups with a treatment being assigned to a tray (90 eggs) at the time of set. The six treatment groups were placed in 3 incubators with two replicate trays per treatment represented within each incubator. The treatments were arranged such that each was represented once randomly in the top half of the incubator and again in the lower half of the incubator (two replicate blocks/incubator) with each of the 3 incubators containing a different random arrangement of those 6 treatments in each of their 2 blocks. Therefore, there were a total of 6 replicate groups (blocks) for each of the 6 treatments across the 3 incubators. The six treatments included a non-injected control (**CNI**), a diluent-injected control (**DT**, Marek's vaccine diluent, Boehringer Ingelheim Animal Health USA INC, Gainesville, GA), a 1X dose of the Poulvac® *E.coli* vaccine (Zoetis, Durham, NC; PECV; 1,000 dose vial resuspended in 25 mL of sterile diluent), a 1 × 10^-2^ dilution, a 1 × 10^-4^ dilution, and a 1 × 10^-6^ dilution of the resuspended PECV.

All eggs were incubated at standard set temperatures (37.5°C dry bulb and 29.76°C wet bulb temperatures between 0 and 18 DOI, and 36.94°C dry bulb and 29.27°C wet bulb temperatures between 18 and 22 DOI). At 12 and 18 DOI, the eggs were candled to verify live embryonation prior to *in ovo* vaccination, and all non-embryonated eggs and eggs with dead embryos were discarded. Mean percent egg weight loss (**PEWL**) for each tray of eggs was calculated between 0 and 12 DOI and between 12 and 18 DOI to establish the lack of an incubational vertical stratification effect on the arrangement of the treatment groups.

### *In ovo* vaccination

Immediately prior to vaccination on 18 DOI, a 1,000 dose vial of PECV was resuspended in 25 mL of sterile diluent (Boehringer Ingelheim Animal Health USA INC, Gainesville, GA). Duplicate plating on LB agar indicated a titer of 2.65 × 10^7^ CFU/ 50 µL dose. Therefore, 10^-2^, 10^-4^, and 10^-6^ dilutions were estimated to contain 2.65 × 10^5^, 2.65 × 10^3^, and 2.65 × 10^1^ CFU/dose, respectively. These *E. coli* doses are hereafter respectively described as 10^7^, 10^5^, 10^3^, and 10^1^ CFU levels. All vaccination solutions were held on ice prior to administration to the eggs.

The *in ovo* vaccination was carried out using an Embrex Inovoject® m injection machine (Zoetis, Durham, NC) set to deliver a 50 μL vaccination volume. The treatments were injected with the diluent treatment first, followed by the lowest to highest *E. coli* doses. The injection process took a total of 1.5 h to complete. Sterile water was flushed through the vaccine line between treatment applications. One egg from each tray (6 eggs from each of the 6 treatments) was removed and injected with Coomassie brilliant blue R-250 dye (MP Biomedicals, LLC. Solon, OH) to identify injection location. Once injection was complete, eggs (including the CNI group) were placed in divided hatch baskets in the 3 incubators during the hatcher phase of the experiment according to the arrangement as described by Elliott et al. (2018) and Fatemi et al. (2023). The arrangement of the treatments was designed to limit possible contamination at hatch, with the lowest to highest doses sequentially placed from the top level to the bottom level of the incubator. The eggs injected with Coomassie blue dye were opened after injection to determine injection site and embryo developmental stage as described by Avakian (2006).

### Hatchery residue analysis, hatchling evaluation, and blood collection at 22 DOI

All handling and care of layer hatchlings was conducted under the approval of the Mississippi State University Institutional Animal Care and Use Committee (Protocol #: IACUC-20-351). All hatchlings were removed from the incubators at 22 DOI. The CNI treatment was removed first, followed by the DT and PECV-injected treatments that were sequentially removed in the order of the least to the greatest dose treatment. The numbers of hatched chicks and residue eggs in each replicate group were recorded. Hatch residue analysis, feather sexing of individual birds, and determination of mean hatchling BW in each treatment-replicate group were then conducted. The embryo residue categories observed from the analysis in each treatment-replicate group at 22 DOI included determination of the percentages of contaminated/rotten eggs (**PCT**); mid-incubation (8 to 13 DOI; **PMD**), and late-incubation (14 to 19 DOI; **PLD**) embryonic deaths; embryos that were dead but had pipped externally (**PDP**); embryos that were found alive in the shell but had not attempted to pip (**PLNP**); and embryos that were alive and had pipped internally (**PLIP**) or externally (**PLEP**). The number of live chicks that had fully hatched was calculated as a percentage of injected eggs that contained live embryos at 18 DOI (**HI**). The percentage chicks that fully hatched but died soon after hatching prior to pull time were also noted.

Five female chicks per treatment in each of the 6 treatments (30 total) were randomly selected for blood collection in 4 mL CAT serum clot activator tubes (Vacuette® Tube. Greiner Bio-One. Monroe, NC) for the purpose of determining serum levels of anti-*E. coli* IgY. These chicks were humanely euthanized by decapitation during blood collection.

### Yolk and body measurements at hatch

From each treatment, 2 female chicks per replicate (72 total) were randomly selected for body length measurements. In an elongated position, body length was measured from the tip of the beak to the end of the extended third toe of the left leg (Joseph et al., 2006; Van den Brand et al., 2019). These birds were then euthanized, weighed individually, and each yolk sac was removed and weighed individually, dried, and weighed again according to the procedures of Peebles et al. (1998). Wet yolk weight was expressed as a percentage of total BW (**PWYW**), and dry yolk weight (**PDYW**) was expressed as percentages of total yolk sac weight. Using the total BW and yolk weight data of the chicks, their yolk-free BW (**YFBW**) was calculated. Furthermore, BW-to-length ratio (**BWTRL**) and percent body mass (**PBM**) were also calculated for each sampled female chick. The BWTLR was calculated by dividing the BW of the chick by its length. Mean PBM was calculated by dividing the yolk-free BW of the chick by its whole BW and multiplying the result by 100.

### Placement of chicks

From the chicks in each of the 6 replicate baskets assigned to each of the 6 treatments, 20 female chicks were randomly selected, and their mean BW was determined before they were placed in each of 6 replicate pens designated for their specific treatment. For placement, the hatching basket treatment-replicate groups that had been established in the incubators were similarly kept in the grow out facility. The grow out facility contained 6 blocks, each containing the 6 main treatments, with a single replicate pen of 20 chicks per treatment represented in each block. The treatments were randomized within each block creating a completely randomized block experimental design. Plastic walls separated individual pens to ensure that no direct contact occurred between birds belonging to different treatment groups and plastic netting covered each pen to prevent bird escape. Birds were fed a non-medicated starter/grower layer diet (Purina® Home Grown® Starter/Grower Complete Feed for Starting and Growing Layer Chickens; Purina Animal Nutrition LLC, Arden Hills, MN). Feed and water were provided for *ad libitum* consumption, and lighting and temperature regimens were employed according to the recommendations of Hy-Line (Hy-Line International, 2020). Bird mortality was monitored twice daily.

All remaining male and female chicks not used in the grow out phase of the study were humanely euthanized via carbon dioxide gas.

### Growout: BW-wk 1 through 3 post hatch

For all treatment groups, the batch BW and numbers of birds in each pen were recorded to determine mean bird BW on 7, 14, and 21 d of growout. The groups of birds in a particular pen were weighed in order of ascending *E. coli* dose treatment (CNI, DT, and then the PECV treatments from lowest to highest *E. coli* dose) to limit bacterial cross-contamination.

Also, on bird weigh dates on 7, 14, and 21 d of growout, from the first block of pens only, 5 randomly selected birds per each treatment (from a single pen in the block) were individually photographed with an infrared image camera (Teledyne FLIR LLC, Willsonville, OR, Model No. FLIR-T1010) to determine the eye temperature of each bird. The specific eye temperature was determined from the image of each photographed pullet using FLIR Thermal Studio software (Version 1.9.3). This data was obtained to test if any of the *in ovo* vaccination dose treatments had any effect on the bird's body temperature, potentially indicating an immune response in the bird to the modified live *E. coli* (Cai et al., 2023).

Blood was collected randomly from 2 birds per replicate pen in each of the 6 treatments (12 per treatment) at 21 days of age for a second determination of serum levels of anti-*E. coli* antibodies. These birds were caught in order of increasing *E. coli* dose treatment. Two additional birds per replicate had their live body lengths and weights measured via the same methods utilized at hatch. Birds that were bled were humanely euthanized by decapitation during blood collection. Blood samples from the birds were collected in 4 mL CAT serum clot activator tubes (Vacuette® Tube. Greiner Bio-One. Monroe, NC). All remaining birds were humanely euthanized.

### Serum ELISA analysis

Serum levels of anti-*E. coli* IgY were determined as previously described (Fatemi et al., 2023). Briefly, 96-well plates were coated with an overnight culture of *E. coli* [ATCC - *Escherichia coli* (Migula) Castellani and Chalmers 25922], diluted 1:10 in ELISA coating buffer (BioRad, Hercules, CA), and incubated overnight at 4°C. Plates were blocked overnight at 4°C with ELISA BSA Block (Bio-Rad, Hercules, CA). Serum samples were diluted 1:500 in blocking buffer, added to the prepared plate in triplicate, and incubated at room temperature for 3 h. Titer levels were determined using goat anti-Chicken IgG conjugated to HRP (BioRad, Hercules, CA) diluted 1:10,000 in blocking buffer and incubated on the plate for 1 h followed by TMB Core+ (BioRad, Hercules, CA) and for 30 min. Two molar H_2_SO_4_ was used as the stop solution. ELISA wash buffer (BioRad, Hercules, CA) was used to wash between each step. Plates were read at 450 nm on an Epoch microplate spectrophotometer (Agilent, Santa Clara, CA). Antibody concentrations were determined from a standard curve developed using unlabeled chicken IgY (SouthernBiotech, Birmingham, AL).

### Statistical analysis

A randomized complete block design was utilized for incubation, hatch and post hatch data. Hatch basket section served as the experimental unit in the analyses of HI, hatch residue egg variables, and mean hatchling BW. Individual embryo and chick data, including body length, whole BW, YFBW, BWTLR, PWYW, percent dry yolk weight, and PBM, chick eye temperatures, and serum ELISA were analyzed with individual bird representing the experimental unit. A two-way ANOVA was utilized to analyze all the data. In the grow out phase, pen served as the experimental unit in the analyses of mean initial, wk 1, wk 2, and wk 3 bird BW. Day 21 body weight and length data were analyzed with individual bird representing the experimental unit.

A two-way ANOVA was used to test for the effects of the *in ovo* injection treatment (fixed effect) and block (random effect) on the incubation and hatch data. Least squares means were compared in the event of significant global effects. Global effects and least squares means differences were considered significant at P ≤ 0.05. All data were analyzed using the MIXED procedure of SAS software 9.4 (SAS Institute, 2013). Treatment means and LS means comparisons for variables that were not significantly affected by treatment are not shown. Fisher's protected least significant difference analysis was performed for treatment means separations (Steel and Torrie, 1980).

The following statistical model was used for the incubation, hatch and post hatch data

$$Y_{ij}=\mu +B_{i}+T_{j}+E_{ij}$$

Where μ was the population mean; B_i_ was the block factor (i = 1 or 6); T_i_ was the effect of each *in ovo* injection treatments (j = 1 to 6); and E_ij_ was the residual error.

Orthogonal polynomial contrasts were also conducted for detecting the dose response effect. The contrasts included the DT treatment group and the dosages of the vaccinated groups. A P ≤ 0.05 was considered significant for a linear, quadratic, or cubic response.

## Results

### Egg weight loss, site of injection, and embryo staging

For sake of conciseness, the means for only those variables that were significantly affected by treatment are shown in Table 1, Table 2. There were no significant differences between treatments for PEWL between 0 and 12 (P = 0.739) and 12 to 18 DOI (P = 0.311). Of the 40 dye injections administered at 18 DOI, 36 (90%) were observed to be successfully administered in the AM. The rest of the injections were observed in either the embryo body (5%) or allantois (5%). The average embryo stage score at 18 DOI was 1.45, which was prior to pipping and prior to the positioning of the head of the embryo under its left wing in preparation for hatch.

**Table 1:** Effects of *in ovo* injection treatments on hatch residue variables, hatchability of injected live embryonated eggs (**HI**), and yolk variables at 22 DOI.


*In ovo* Injection Treatments^1^	PLD^2^^,^^3^	PLIP^2^^,^^3^	PLEP^2^^,^^3^	HI^3^	PWYW^4^	PDYW^4^
	**———————————(%)———————————**					
CNI	1.79^c^	0.41^c^	1.19^cd^	96.0^a^	8.90^b^	50.1^a^
DT	5.00^b^	0.21^c^	1.03^d^	93.8^ab^	10.58^ab^	50.7^a^
2.65 × 10^1^	4.33^bc^	1.01^bc^	4.30bcd	89.3^bc^	9.55^b^	47.8^b^
2.65 × 10^3^	4.10^bc^	1.41^bc^	5.42^bc^	87.5^c^	12.52^a^	48.4^ab^
2.65 × 10^5^	4.55^bc^	1.84^b^	7.09^b^	85.3^c^	11.03^ab^	47.1^b^
2.65 × 10^7^	8.07^a^	4.97^a^	15.64^a^	68.2^d^	12.62^a^	48.9^ab^
Pooled SEM	1.09	0.55	1.99	2.35	1.08	0.009
P-value	0.011	<0.0001	<0.0001	<0.0001	0.047	0.026
Polynomial Contrasts P Values						
Linear	0.005	<.0001	<.0001	<.0001	0.125	0.155
Quadratic	0.978	0.084	0.054	0.039	0.891	0.775
Cubic	0.661	0.159	0.139	0.090	0.045	0.087

a-d Means in the variable column with no common superscript differ significantly (P ≤ 0.05).

1 CNI = non-injected control treatment. DT = Marek's disease commercial diluent-injected control treatment. Poulvac *E. coli* vaccine CFU administered per egg are designated as: 2.65 × 10^1^, 2.65 × 10^3^, 2.65 × 10^5^, and 2.65 × 10^7^ CFU/egg (50 µL dose).

2 Percentages of late-incubation (14-19 DOI) embryonic deaths (**PLD**); and live embryos that pipped internally (**PLIP**) or externally (**PLEP**) but that did not complete hatch.

3 N = 6 replicate hatching basket sections in each treatment, with each hatching basket section containing a maximum of 90 eggs.

4 N = 12 replicate chicks per treatment. PWYW = wet yolk sac weight as a percentage of the individual chick BW; PDYW = the dried yolk sac weight as a percentage of the individual chick BW.

**Table 2:** Effects of *in ovo* injection treatment on mean female chick BW at 0 (placement), 7, 14, and 21 d, and body length at 21 d post hatch.

*In ovo* Injection Treatments^1^	BW-0 d^3^	BW-7 d^3^	BW-14 d^3^	BW-21 d^3^	Body Length-21 d^4^
	————————–(g)————————–				cm
CNI^1^	38.6	73.6^b^	127.9^b^	193.3^b^	31.6^a^
DT	39.4	75.9^a^	131.5^a^	199.7^a^	31.6^a^
2.65 × 10^1^	38.6	72.3^b^	124.7^bc^	190.7^bc^	31.2^ab^
2.65 × 10^3^	38.4	70.2^c^	123.1^c^	187.3^cd^	31.2^ab^
2.65 × 10^5^	38.3	68.9^c^	117.8^d^	183.0^de^	31.1^ab^
2.65 × 10^7^	38.6	65.7^d^	115.1^d^	180.6^e^	30.7^b^
Pooled SEM	0.49	0.67	1.19	1.59	0.22
P-value	0.308	<0.0001	<0.0001	<0.0001	0.045
Polynomial Contrasts P Values					
Linear	0.717	<.0001	<.0001	<.0001	0.038
Quadratic	0.238	<.0001	<.0001	<.0001	0.320
Cubic	0.145	<.0001	0.0019	0.0004	0.441

a-e Means in the variable column with no common superscript differ significantly (P ≤ 0.05).

1 CNI = non-injected control treatment. DT = Marek's disease commercial diluent-injected control treatment. Poulvac *E. coli* vaccine CFU administered per egg are designated as: 2.65 × 10^1^, 2.65 × 10^3^, 2.65 × 10^5^, and 2.65 × 10^7^ CFU/egg (50 µL dose).

3 N = 20 birds in each of 6 replicate pens in each treatment.

4 N = 2 birds in each of 6 replicate pens in each treatment.

### Residue eggs and hatchability

Mean PCT (P = 0.435), PMD (none observed), PDP (P = 0.056), and PLNP (P = 0.053) were not significantly affected by treatment. Mean PLD was significantly (P = 0.011) affected by treatment (Table 1). Mean PLD was higher in the 10^7^-treatment compared to all other treatments. Mean PLD was higher in the DT treatment when compared to the CNI treatment, with the 10^1^, 10^3^, and 10^5^ treatments intermediate. The PLD also showed a significant linear effect with the increasing dosage of the PECV delivered *in ovo*.

Mean PLIP and PLEP were significantly (P < 0.0001) affected by treatment (Table 1). There was a significantly higher PLIP value in the 10^7^-treatment in comparison to all other treatments. In the 10^5^-treatment, there was a higher PLIP value when compared to the CNI and DT treatments, with the 10^1^ and 10^3^ treatments being intermediate. The PLIP showed a significant linear effect with increasing dosage of the *in ovo* PECV. There was a significantly higher PLEP in the 10^7^-treatment in comparison to all other treatments. In the 10^5^-treatment, there was a higher PLEP than in the CNI treatment, with the 10^3^-treatment being intermediate. Furthermore, there was a higher PLEP in the 10^3^-treatment than in the DT treatment, with the CNI and 10^1^ treatments being intermediate. The PLEP also had a significant linear effect with a marginally significant quadratic effect.

Mean HI was significantly (P < 0.0001) affected by treatment (Table 1). The HI in the CNI treatment was higher when compared to the 10^1^, 10^3^, 10^5^, and 10^7^ treatments, with that in the DT treatment intermediate. In the DT treatment, there was a higher HI compared to the 10^3^, 10^5^, and 10^7^ treatments, with the HI of the 10^1^-treatment being intermediate. There was also a higher HI in the 10^1^-treatment compared to that in the 10^7^-treatment, and those in the 10^3^ and 10^5^ treatments were also higher than that in the 10^7^-treatment. The HI polynomial contrasts including the DT and the dosages of the *in ovo* vaccine had a significant linear effect with the decrease in hatch seen with the increase in vaccine dosage as well as a significant quadratic effect. There was no significant difference between the treatments for percentage chicks that fully hatched but died soon after hatching prior to pull time (P= 0.435) or for mean hatchling BW at 22 DOI (P = 0.053).

### Yolk and body measurements at hatch

For the 2 hatchlings selected from each main treatment-replicate group, mean whole BW (P = 0.238), YFBW (P = 0.212), wet yolk weight (P = 0.083), dry yolk weight (P = 0.164), body length (P = 0.070), BWTLR (P = 0.386), and PBM (P = 0.116) of the female chicks at 22 DOI were not significantly different between treatments.

Mean PWYW of the female chicks was significantly (P = 0.047) affected by treatment (Table 1). Mean PWYW of the chicks in the 10^3^ and 10^7^ treatments was higher than those in the CNI and 10^1^ treatments, with that in the DT and 10^5^ treatments being intermediate. The PWYW had a significant cubic effect. Mean PDYW were also significantly (P = 0.026) affected by treatment (Table 1) but showed no significant linear, quadratic, or cubic effects. Mean PDYW of the chicks in the CNI and DT treatments was significantly higher than those in the 10^1^ and 10^5^ treatments, with the PDYW of chicks in the 10^3^ and 10^7^ treatments being intermediate.

### Bird mortality

Over the 3 wk course of the grow out period, a total of 6 birds died, all from the 10^7^-treatment. All 6 birds excreted watery feces and exhibited external signs of dehydration. Upon necropsy, all had internal signs of infection, with ballooned intestines and yellow purulent drainage lining the peritoneum of the body cavity. Cumulative percent mortality of the birds in the 10^7^-treatment over the 3 wk post hatch period was 5.41%.

### Grow out bird body weight, length, and infrared eye temperature

Mean chick BW at placement was not significantly different between treatments (P = 0.308). Although not significant, these data are included in Table 2 for baseline comparison with the subsequent BW means. However, mean chick BW at 7, 14, and 21 d of grow out was significantly (P < 0.0001) affected by treatment (Table 2). At 7 d of grow out, mean BW of female chicks was significantly higher in the DT treatment compared to all other treatments. The BW of the chicks in the CNI and 10^1^ treatments was significantly higher than those in the 10^3^, 10^5^, and 10^7^ treatments, and those in the 10^3^ and 10^5^ treatments were higher than those in the 10^7^-treatment. At 14 d of grow out, mean BW of female chicks was significantly higher in the DT treatment when compared to all other treatments. The BW of those in the CNI treatment was higher than those in the 10^3^-treatment, with that in the 10^1^-treatment being intermediate. The BW of those in the 10^3^-treatment was higher than those in the 10^5^ and 10^7^ treatments. At 21 d of grow out, mean BW of female chicks was significantly higher in the DT treatment, when compared to all other treatments. The BW of those in the CNI treatment was higher than those in the 10^3^-treatment, with the BW of those in the 10^1^-treatment being intermediate. The BW of those in the 10^3^-treatment was higher than those in the 10^7^-treatment, with those in the 10^5^-treatment being intermediate. The average bird BW at 7, 14, and 21 d all showed significant linear, quadratic, and cubic effects.

The mean BW (P = 0.437) and BWTLR (P = 0.643) of chicks individually measured at 21 d of grow out were not significantly affected by treatment. However, chick body length at 21 d of grow out was significantly (P = 0.045) affected by treatment (Table 2). Chick body length was significantly higher in those in the CNI and DT treatments when compared to those in the 10^7^-treatment, with those the 10^1^, 10^3^, and 10^5^ treatments being intermediate. Chick body length at 21 d showed a significant linear effect decreasing with increasing vaccine dosage.

The eye temperatures measured with the thermal camera at 7, 14, and 21 d were not affected by the *in ovo* vaccination treatment at any of the measured ages (P = 0.571 at 7d; P = 0.408 at 14d; and P = 0.307 at 21 d).

### Blood analysis

Anti-*E. coli* IgY was prevalent in the sampled newly hatched chicks with percentage positive chicks being 80% for the 5 CNI chicks, 60% in the DT, 100% in the 10^1^ treatment, 60% in the 10^3^ treatment, 60% in the 10^5^ treatment, and 40% in the 10^7^ treatment. The antibody concentration (ng/mL) from the positive chicks at hatch showed no significant difference between the 6 treatments (P = 0.175). Anti-*E. coli* IgY was not detected in serum of any birds at three weeks of age (P > 0.05).

## Discussion

There were no significant differences between treatments for PEWL between both the 0 to 12 and 12 to 18 DOI periods, indicating that the eggs in all the treatment groups shared similar incubational environments, thereby ensuring that the noted effects of treatment on the variables examined were not confounded by environmental influences during incubation.

In the current study, 36 of the 40 examined AM injections were successfully administered in the AM. According to Avakian (2006), embryos injected at 18 DOI with an Inovoject machine have a greater than 90% chance of their injections occurring in the AM, with approximately 5.79% of their injections occurring in the allantoic sac, air cell, or yolk sac. The current results of this and previous studies indicate that a majority of the injections administered at 18 DOI were successfully administered in the AM (Sokale et al., 2018; Elliott et al., 2022; Fatemi et al., 2023).

The aim of the current study was to examine not only the effects of the *in ovo* injection of wider doses of the PECV on hatch success, but also the subsequent effects on post hatch live performance and the serological response of layers. In comparison to the CNI and DT control groups, injection of the PECV in the 10^3^, 10^5^, and 10^7^ treatments resulted in a significant reduction in HI, with the 10^1^-treatment being intermediate. It is worth noting that when compared to the significant decrease in hatchability in response to *E. coli* doses between 4.4 × 10^2^ and 4.4 × 10^8^ CFU, as noted by Fatemi et al. (2023), the 10^1^ dose in this study did not lead to a significant decrease in HI, indicating that an increase in *E. coli* dose between 10^1^ and 10^2^ CFU may be the point at which *E. coli* administered via the AM at 18 DOI becomes significantly detrimental to the layer embryo. In addition, the highest dose (10^7^ CFU) led to a further reduction in HI in comparison to the other 3 lower doses. These results are similar to the *in ovo* injection of various doses of F-strain of *Mycoplasma gallisepticum* in Hy-Line W-36 layer embryos at 18 DOI (Elliott et al, 2017) in which a full dose as well as 1 × 10^-2^, 1 × 10^-4^, and 1 × 10^-6^ dilutions of the Poulvac Myco F vaccine were used. It was found that HI decreased as dose increased, with an approximate 60% HI observed in the full dose treatment (Elliott et al., 2017). Thus, these comparable results indicate that *in ovo* vaccination with a modified/attenuated live bacterial vaccine in the amnion require low doses of bacteria (CFU/egg) to have the least effect on hatchability.

The 10^7^-treatment had a significantly higher level of PLD (14 to 19 DOI) embryos in comparison to all other treatments. In addition, mean PLIP and PLEP were significantly higher in the 10^7^-treatment in comparison to all other treatments. A similar pattern was also observed with AM *in ovo* injection of PECV in the studies by Fatemi et al. (2023). These results indicate that when injected at 18 DOI in the AM, the *E. coli* of the PECV at the doses delivered in this study, especially at the highest dose, compromised late embryonic viability, thereby impairing the ability of the embryo to complete a normal hatching process. The increase in PLD, PLEP, and PLIP were evidently the major cause of the significant decrease in HI in response to the highest *E. coli* doses. Elliott et al. (2017) also reported a lower hatch of embryonated eggs in the higher *Mycoplasma gallisepticum in ovo* injection treatment groups. More specifically, there was a significantly higher percentage of embryos that died while pipping after receiving a medium (1 × 10^4^ CFU) or full (1 × 10^6^ CFU) dose of the Poulvac Myco F Vaccine. Triplett et al. (2018) administered 3 different probiotic strains of bacteria (*Lactobacillus acidophilus, Bacillus subtillus*, and *Bifidobacterium animalis*) by *in ovo* injection to broiler hatching eggs at 18 DOI. In the *Bacillus subtillus* treatment, there was a significant increase in the percentage of embryos that died during the pipping process. Upon consideration of various possible reasons for the increase in embryos that died while pipping, Triplett et al. (2018) suggested that the embryos in that treatment group may have lacked sufficient energy reserves to immunologically resist the bacterial challenge. This same type of response to the *E. coli* bacterial challenge in the current study may also have occurred. In the current study, there was no statistical difference in hatch percentage between the CNI and the DT, however, there was a statistically greater PLD in the DT treatment than in the CNI treatment. The greater PLD was not significantly different between the DT, the 10^1^, 10^3^, and 10^5^ treatments with only the 10^7^-treatment having the highest PLD. This difference may be influenced by the *in ovo* injection itself, although this difference has not been seen in previous studies (Elliott et al., 2018; Fatemi et al., 2020b).

By 18 DOI, chicken embryos are capable of activating an immune response against pathogens they come into contact with (Hincke et al., 2019). However, immune competence tends to increase with age, with older birds having a better immune response to pathogens than embryos or young chicks (Alkie et al., 2019). An increase in mortality is not seen when the PECV is administered via commercial coarse spray to chicks at one day of age (Christensen and Nielsen, 2020; Zoetis, 2021a). It is likely that only an innate immune response was activated when the embryos were vaccinated at 18 DOI, and the response was likely not strong enough to overcome the large amounts of *E. coli* deposited in the AM. This might be a possible explanation as to why increases in PLD, PLIP, and PLEP were observed when the PECV was administered via *in ovo* injection.

Maternal antibodies, transferred from the breeder hen to the embryo via the egg yolk, are a chick's leading defense against pathogens until they can produce their own immune cells (Hincke et al., 2019). The hens that laid the hatching eggs used in the current study were vaccinated for *E. coli* and, according to the serum ELISA results, conveyed their antibodies to the resulting progeny at hatch. In a study conducted by Weber, SPF chicks were challenged with APEC at either 7, 14, or 21 d of age (Poultry Health Today, 2020). Blood samples were taken on day of hatch to test for the presence of maternal antibodies in chicks that later served as unvaccinated controls or that were vaccinated at one day of age with the PECV. While it was found that the SPF chicks did indeed carry maternal antibodies for *E. coli*, it was also concluded that the presence of maternal antibodies alone were not sufficient in protecting chicks from an APEC infection. While control chicks showed a high percentage of colibacillosis lesions at 7, 14, and 21 d of growout, chicks that had been vaccinated with the PECV had a significantly lower presence of lesions at 14 and 21 d of growout (Poultry Health Today, 2020). This further indicates that immune function increases with age. The embryos in the current study received maternal antibodies against *E. coli*, however, the levels of antibodies that they possessed may not have been sufficient enough to protect them from the large amounts of *E. coli* vaccine administered at 18 DOI in the higher doses tested.

Mean PWYW at 22 DOI was significantly higher in the chicks in the 10^3^ and 10^7^ treatments. This indicates that the hatched chicks from the PECV treatment groups retained heavier yolk sacs. Having a larger residual yolk could be another result due to the injection of the PECV. A mounted immunological resistance to the bacterial insult could have led to a reduction in energy utilization, thus causing the affected chicks to retain more yolk. The YFBW in this trial, however, was not affected by the *in ovo* vaccination treatment. The PDYW, or percentage solid portion of the yolk sac statistically did not show elevated solids in the 10^7^ and 10^3^ treatments in comparison to the two control treatments (CNI and DT). These results, however, differ from those of Elliott et al. (2017), who found no significant differences in YFBW, WYW, PWYW, DYW, or PDYW between controls and embryos *in ovo*-vaccinated with the Poulvac Myco F vaccine at 18 DOI. Nevertheless, the results of the current study were similar to the results reported in a study by Montgomery et al. (1999), who injected Ec^NAL^, an adapted form of *E. coli*, into the chorioallantoic sac of broiler embryos at 12 DOI. Of the chicks that hatched, it was discovered that the chicks injected with Ec^NAL^ had heavier yolks and a higher yolk mass relative to body mass when compared to control groups. Furthermore, Fatemi et al. (2020b, 2020a) reported that an increase in percentage of yolk moisture was associated with impaired post hatch performance, and therefore the negative results in body weight and length during the growing phase could be due in part to the yolk characteristics observed for the PECV treatments as compared to the control groups.

Mean BW at 7, 14, and 21 d of grow out decreased with an increase in PECV dose, with doses at or above 10^3^ CFU significantly decreasing post hatch growth. Like the comparison of hatchability rates observed in this study and that by Fatemi et al. (2023), it is suggested that an increase in *E. coli* dose between 10^1^ and 10^2^ CFU may be the point at which *E. coli* administered via the AM at 18 DOI also becomes significantly deleterious to post hatch growth, with no recovery through 21 d of grow out. An *E. coli* infection has been shown to decrease BW in birds during the growout phase. Gross (1964) used White Leghorn embryonated hatching eggs in an experiment in which they were dipped for 20 sec. at 18 DOI in an APEC solution that contained 1 × 10^7^ CFU of *E. coli*. Between wk 3 and 9 of grow out, birds that had been exposed to the *E. coli* at 18 DOI had a lower BW (Gross, 1964). Similar results were seen in the previously mentioned study by Montgomery et al. (1999). In that study, in birds which received EcNAL into the chorioallantoic sac at 12 DOI and were reared until 21 d of age, it was found that the mean BW of the chicks that were exposed to EcNAL had a significantly lower mean BW in comparison to those in the control groups (Montgomery et al., 1999). It is possible that, in the current study, an embryonic exposure to *E. coli* in the PECV led to the decreased post hatch BW of birds that belonged to the 10^1^, 10^3^, 10^5^, and 10^7^ treatment groups.

The chicks in the CNI and DT treatments were also observed to have significantly longer body lengths at 21 d of grow out compared to those in the 10^7^-treatment, with those in the other 3 PECV treatments being intermediate. Chick body length has been viewed as a variable that can be a predictor of later chick quality, with a longer body length being associated with improved post hatch performance (Molenaar et al., 2008). It is evident that a full dose of the PECV at 18 DOI decreases chick quality.

When comparing the CNI and DT treatments post hatch, the two control treatments had no difference in BW at hatch, but the DT treatment maintained a significantly greater BW at 7, 14, and 21 d. The CNI and DT treatments did not differ in body length at 21 d. There is potential that the diluent itself delivered *in ovo* may have a beneficial effect on the growth of the layer chicken, however, this finding has not been confirmed in previous studies with commercial layer chickens (Elliott et al., 2018, 2022).

The chicks in the current study were removed from the hatcher and placed in the house in order from the CNI and DT chicks then with increasing PECV dosage until the highest dosage treatment was placed with access to feed. The time from the placement of the CNI treatment group to the final placement of the 10^7^-treatment group was 1.5 h. Previous research has shown that earlier access to feed results in improved BW post hatch, however, previous studies have evaluated more extreme timing of delayed feeding than 1.5 h (Noy and Skylan, 1999; Bigot et al., 2003). To our knowledge, we are not certain of a BW effect of a 1.5 h delay in feeding for newly hatched chicks, however, this should be considered for a potential additional factor in the post hatch BW differences seen in the current study.

In the current study, serum antibody anti-*E. coli* IgY levels were tested at hatch and at 21 d post hatch. The prevalent finding of maternal antibodies in all treatments may be due to the hen's vaccination and/or natural immune response to *E. coli* in the environment (Poultry Health Today, 2020). The lack of any serum antibody anti-*E. coli* IgY at 21 d is not surprising given the aroA deletion in the PECV that does not allow persistence of the bacteria within the bird and initiation of the bird's own antibody production (Salehi et al., 2012). A cellular response, as opposed to a humoral antibody response, is believed to be the reason for the success of day of age spray vaccination, which offers protection to the bird (Filho et al., 2013). This may additionally explain the lack of body temperature differences (measured via eye temperature) in the current study. The bird's immune system may have only activated a cellular response, and the lack of persistence of the bacteria with the aroA deletion (lack of infection) did not cause any elevated body temperature responses in the birds. Future study on the *in ovo* vaccination of PECV would need to include evaluations of its protection against an APEC challenge and the potentiality of it eliciting a cellular immune response in the bird.

In conclusion, it was shown in this study that the PECV can be successfully delivered to the AM of Hy-Line W-36-layer hatching eggs at 18 DOI. Nevertheless, the 18 DOI injection of the PECV into the AM containing *E. coli* doses at or above 10^3^ CFU, particularly 10^7^ CFU, may incur detrimental effects on late embryo livability and subsequent HI, and on the livability and growth of layer pullets through 3 wk post hatch. Future research should include evaluations of the immunological protection afforded by the *in ovo* vaccination of PECV prior to an *E.coli* challenge and any subsequent financial benefits it may provide. The investigation of additional sites of vaccination, such as the air cell, may also be beneficial.

## Disclosures

The authors declare the following financial interests/personal relationships which may be considered as potential competing interests:

Katie Elliott reports equipment, drugs, or supplies was provided by Boehringer Ingelheim. Katie Elliott reports equipment, drugs, or supplies was provided by Zoetis Animal Health Co. If there are other authors, they declare that they have no known competing financial interests or personal relationships that could have appeared to influence the work reported in this paper.
